# Application of magnetic nanoparticles in nucleic acid detection

**DOI:** 10.1186/s12951-020-00613-6

**Published:** 2020-04-21

**Authors:** Congli Tang, Ziyu He, Hongmei Liu, Yuyue Xu, Hao Huang, Gaojian Yang, Ziqi Xiao, Song Li, Hongna Liu, Yan Deng, Zhu Chen, Hui Chen, Nongyue He

**Affiliations:** 1 Hunan Key Laboratory of Biomedical Nanomaterials and Devices, Hunan University of Technology, Zhuzhou, 412007 China; 2 State Key Laboratory of Bioelectronics, Southeast University, Nanjing, 210096 China

**Keywords:** Magnetic nanoparticles, Nucleic acid detection, High-throughput sequencing, Clinical diagnosis, Magnetic separation

## Abstract

Nucleic acid is the main material for storing, copying, and transmitting genetic information. Gene sequencing is of great significance in DNA damage research, gene therapy, mutation analysis, bacterial infection, drug development, and clinical diagnosis. Gene detection has a wide range of applications, such as environmental, biomedical, pharmaceutical, agriculture and forensic medicine to name a few. Compared with Sanger sequencing, high-throughput sequencing technology has the advantages of larger output, high resolution, and low cost which greatly promotes the application of sequencing technology in life science research. Magnetic nanoparticles, as an important part of nanomaterials, have been widely used in various applications because of their good dispersion, high surface area, low cost, easy separation in buffer systems and signal detection. Based on the above, the application of magnetic nanoparticles in nucleic acid detection was reviewed.

## Background

Magnetic nanoparticles (MNPs) have the characteristics of both magnetic particles and nanoparticles (NPs), which refer to particles ranging between 1 and 100 nm that produce a response when presented in magnetic field. As the size of MNPs decrease, the ratio of surface area to volume increases allowing an increase with surface effect, small size effect, quantum size effect and macroscopic quantum tunneling effect. MNPs also exhibit coercivity changes and a Curie temperature decrease [1, 2].

MNPs are classified into metal NPs, metal oxide NPs and alloy NPs. Metal NPs includes iron, cobalt, and nickel [3]. Metal oxide NPs consist of iron oxides (γ-Fe_2_O_3_ and Fe_3_O_4_) and ferrites (CoFe_2_O_4_ and Mn_0.6_Zn_0.4_Fe_2_O_4_) [4]. Alloy NPs include FeCo and FePt [5].

Some magnetic nanoparticles have superparamagnetism, which refers to the state of MNPs when introduced to an external magnetic field, in which NPs react similarly to paramagnets with the difference being the higher level of attraction, hence “super”. All the MNPs are easily guided in the presence of external magnetic fields [6]. This is often used in materials science, electrochemistry, biochemical sensing, magnetic resonance imaging (MRI), environmental and medical research [7]. MNPs also play a significant role in removing pollutants and relieving toxicity, such as membrane separation for water treatment and purification. Researchers use MNPs to immobilize biomolecules (antibodies, proteins, enzymes, etc.) and utilize simple, rapid, cheap and efficient separation of target biomolecules [8–15]. Biomarkers in complex clinical samples can be preconcentrated and enriched, separating interfering matrices, and increasing the sensitivity and specificity of testing. Magnetic drug targeting utilizes drug targeted delivery in vivo through active targeted therapy strategy. In vitro detection and application of MNPs plays an important role in early rapid diagnosis and treatment of diseases, thus assisting in prevention, management, treatment and prognosis of diseases [16–23]. MNPs are also used for lateral flow test strips and microfluidic platforms such as lab-on-a-chip (LOC) devices for continuous flow of magnetic cell separation, and can be developed into portable devices that are easy to use. MNPs have attracted great interest of researchers due to their excellent properties [24–31].

Nucleic acid is one of the most basic substances in life and has extremely important biological functions. It mainly stores and transmits genetic information [32–36] which in turn, helps detect genetic changes that may be associated with certain health conditions [37–41]. The following is a brief review outlining the significance of nucleic acid detection in identifying important gene mutations for disease risk prediction, clinical treatment and prognosis evaluation [42–48]. Research shows that breast cancer susceptibility gene mutations are the main cause of breast cancer family clustering [49–54]. Berenstein [55] studies show that FLT3 gene mutation is one of the most frequent gene mutations in patients with Acute myeloid leukemia (AML), which can be seen in various subtypes of AMI. Kayser [56] found that C-KIT mutations are present in leukemia patients. Through gene sequencing, it can be seen that lung cancer-related genes such as epidermal growth factor receptor gene mutation, c-MET, ROS1, KRAS, and BRAF, play an extremely important role in diagnosis and treatment of lung cancer [57–71]. In addition to the above diseases, many diseases are related to gene mutations, such as Type 1 diabetes mellitus [72–75] and Type 2 diabetes mellitus [76–81], Lymphoma [82, 83], bowel cancer [84–88], and prostatic cancer [89–91]. Early nucleic acid detection is significant for identifying these gene mutations to prevent, treat, and identify disease prognosis [92].

To date, the gold standard for nucleic acid detection is polymerase chain reaction (PCR), however, this method is both time-consuming and laborious. PCR detection equipment is both large and expensive, and the operators need professional training. Therefore, it is especially important to develop fast and economical nucleic acid detection technology and equipment. The conventional nucleic acid extraction and isolation is a time-consuming process that goes through several centrifugation steps often resulting in low yield and purity. The above limitations restrict the application of such technologies for real time testing, limiting PCR to more developed central cities, hospitals and medical institutions in developed countries. PCR consumables and machines are often too expensive for low-level medical service platforms, especially in the vast rural areas and medical institutions in developing countries. In addition, the traditional PCR-based detection methods have been difficult to meet the increasing high-throughput demand in recent years for emergency treatment of sudden infectious diseases, clinical in vitro diagnosis, mobile medicine and other applications due to time restraints.

MNPs have high surface area to volume ratio, high binding rate with detection substances, and can perform magnetically controllable aggregation and dispersion, making preconcentration, purification and separation of nucleic acids simple and easy. MNPs have good dispersibility, which can bind biomolecules quickly and effectively. The binding is reversible, and the aggregation and dispersion of MNPs can be controlled. In the absence of an external magnetic field, the particles are nonmagnetic and are uniformly suspended in solution, while when an external magnetic field is used, the particles have magnetism and can be separated. Active substances such as bioactive adsorbents or other ligands connected to the surface of MNPs can be combined with specific biomolecules, like enzymes, DNA, proteins, and separated under the action of an external magnetic field. This method has high specificity, rapid separation and good reproducibility. MNPs have been applied in biosensors to improve the sensitivity of nucleic acid detection, and have been widely used in nucleic acid detection due to their excellent properties. A series of automatic detection instruments have been designed to utilize rapid and automatic detection of nucleic acid, which is of great significance in the medical field [93–99].

This article introduces the application of MNPs in nucleic acid extraction, target enrichment, infectious disease identification, site mutation detection, and library preparation for Next Generation Sequencing.

## Nucleic acid extraction

Nucleic acid extraction is one of the most fundamental steps in molecular biology applications and is a prerequisite for many experiments. The quality of extracted nucleic acid will directly affect the results of subsequent detection in experiments. Traditional nucleic acid extraction methods are both time-consuming and laborious, in addition to operators requiring professional training [100]. Magnetic beads (MBs) are considered a powerful tool for nucleic acid extraction. Coupling of magnetic properties with specific ligands in MBs allows the separation and purification of nucleic acids in a highly efficient and specific manner. In fact, this technique has driven a technological revolution in biological research [101]. Pang [102] proposed a functionalized Fe_3_O_4_@Ag magnetic nanoparticle biosensor to capture and ultra-sensitively detect microRNA from total RNA of cancer cells. Chen [103] developed a paper-based analytical device (PAD) for DNA colorimetric detection, which detected the specific recognition of nucleic acids through MNP separation and hybridization chain reaction (HCR) induced by target DNA.

Wang [104] used MNPs to simultaneously extract DNA and RNA from cancer cells. This MNP-based method has attracted great attention because of its convenient operation, low cost and easy automation. In order to extract nucleic acids efficiently and programmatically, in Li’s [105] research, a rapid and high-quality universal nucleic acid extraction kit based on MBs was developed and named MB-100.

Wu [106] demonstrates the ease of MB automation by designing a new type of automatic sample processing device based on magnetic separation to isolate and extract both DNA and RNA. This device enables sample preparation to be completed in the field without transferring the plate to any additional equipment. Similarly, Chen [107] developed an automatic rapid nucleic acid extractor, which can process 16 samples simultaneously using MNPs. The nucleic acid extraction system can complete the whole nucleic acid extraction process within 30 min, and the system is stable and reliable. Recently, Tavallaie [108] proposed that the recombination of gold-plated MNP networks modified by probe DNA (DNA-Au @ MnPs) induced by electric field can construct a highly sensitive sensor for analyzing nucleic acids in complex samples such as whole blood.

The method of extracting nucleic acids by MBs is widely used in various fields. Applying a magnetic field attracts the target-bound molecule towards the magnet, separating them from the unwanted material or inhibitors without disturbing the nucleic acid of your interest. The method of extracting nucleic acids by magnetic beads has become the mainstream of modern molecular biology. Table 1 details extraction applications mentioned in this paper. A major advantage of MBs application in extraction is that the aggregations do not need centrifugation, which can significantly ease the test process and equipment requirements, thus improving efficiency and lowering cost.

**Table 1:** A summary of extraction

Magnetic particles	Experimental sample	Extraction target	References
Fe_3_O_4_@Ag	HeLa, A549, MCF-7	microRNA	[102]
Fe_3_O_4_@SiO_2_	Hep G2	DNA/RNA	[104]
MBs@SiO_2_	E. coli, breast cancer	DNA	[105]
MNPs	*E. coli*	DNA	[106]
MBs	Liver cancer cells, *E. coli*	DNA	[107]
Au@MNPs	A549	microRNA	[108]

## Enrich target substance

Using MNPs for target enrichment capture technologies allow scientists to rapidly interrogate important genomic regions of interest for variant discovery including infectious disease detection, Single Nucleotide Polymorphism (SNP), gene isoforms, and structural variation which highly improves the accuracy and sensitivity assays and greatly reduces the experimental cost and turnaround time.

**Fig. 1:**
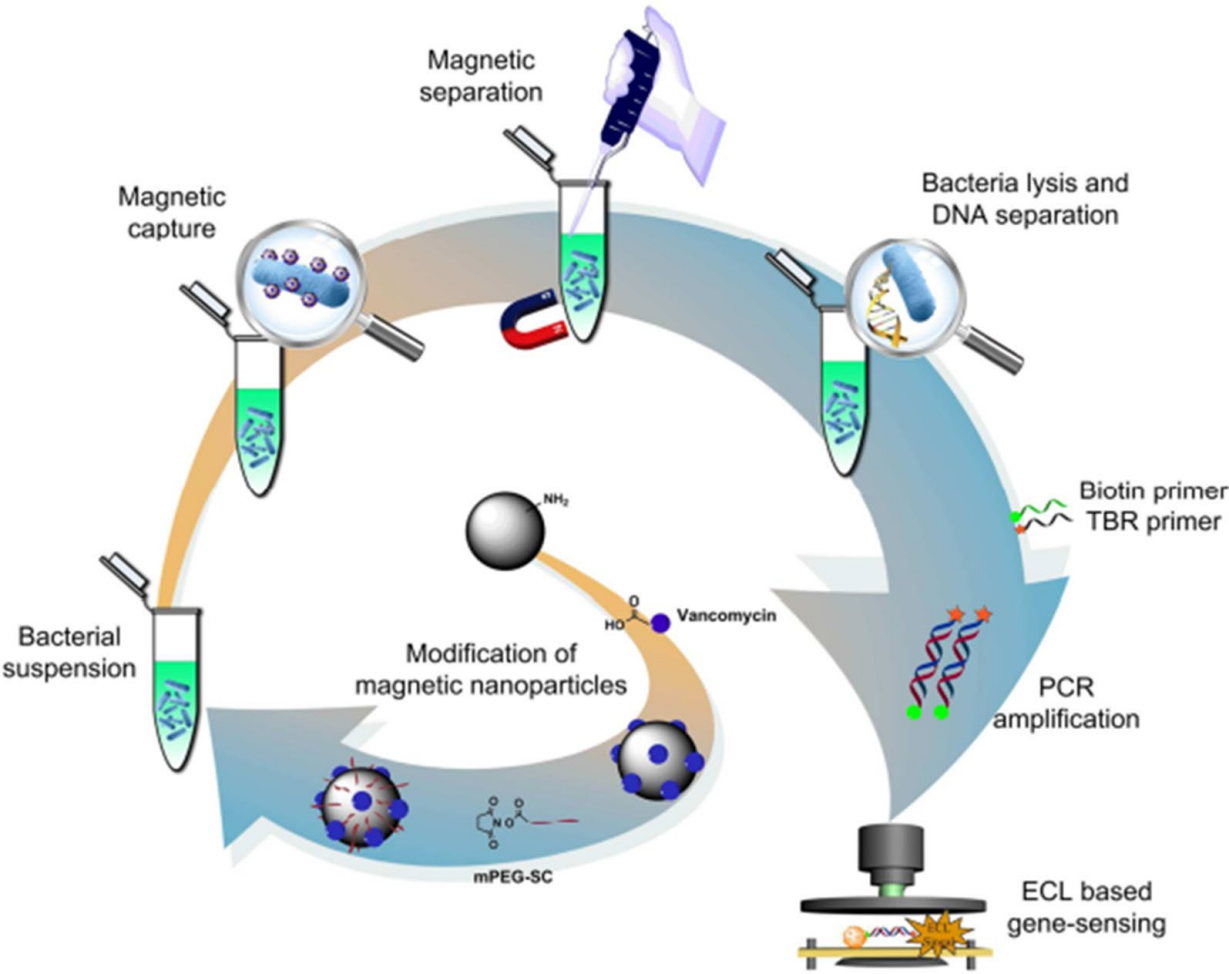
The schematic procedure of the integrated bacterial enrichment/gene-sensing system with Fe_3_O_4_/vancomycin/PEG nanocarrier [109]

**Fig. 2:**
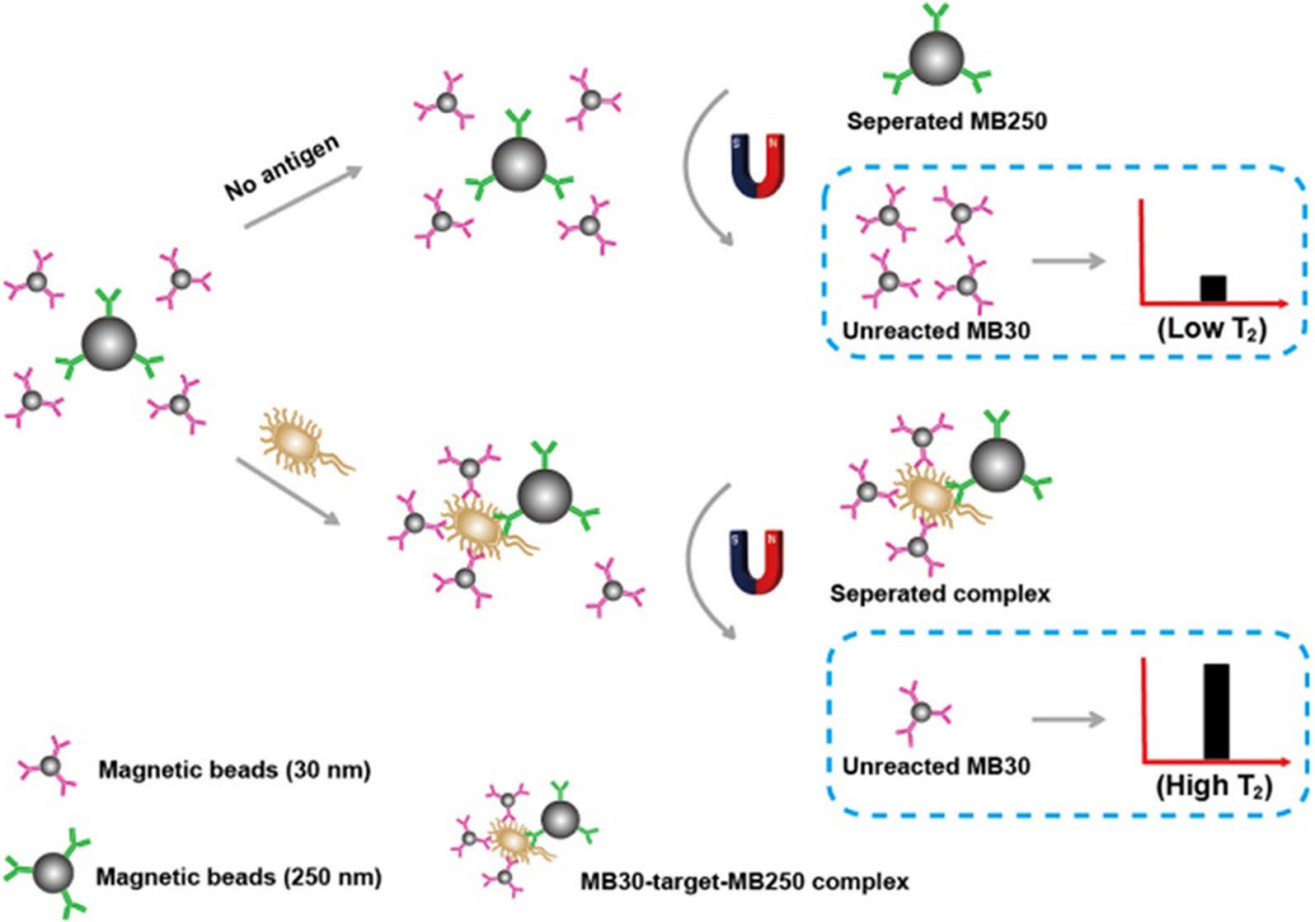
Schematic illustration of the MS-MRS sensor [110]

**Fig. 3:**
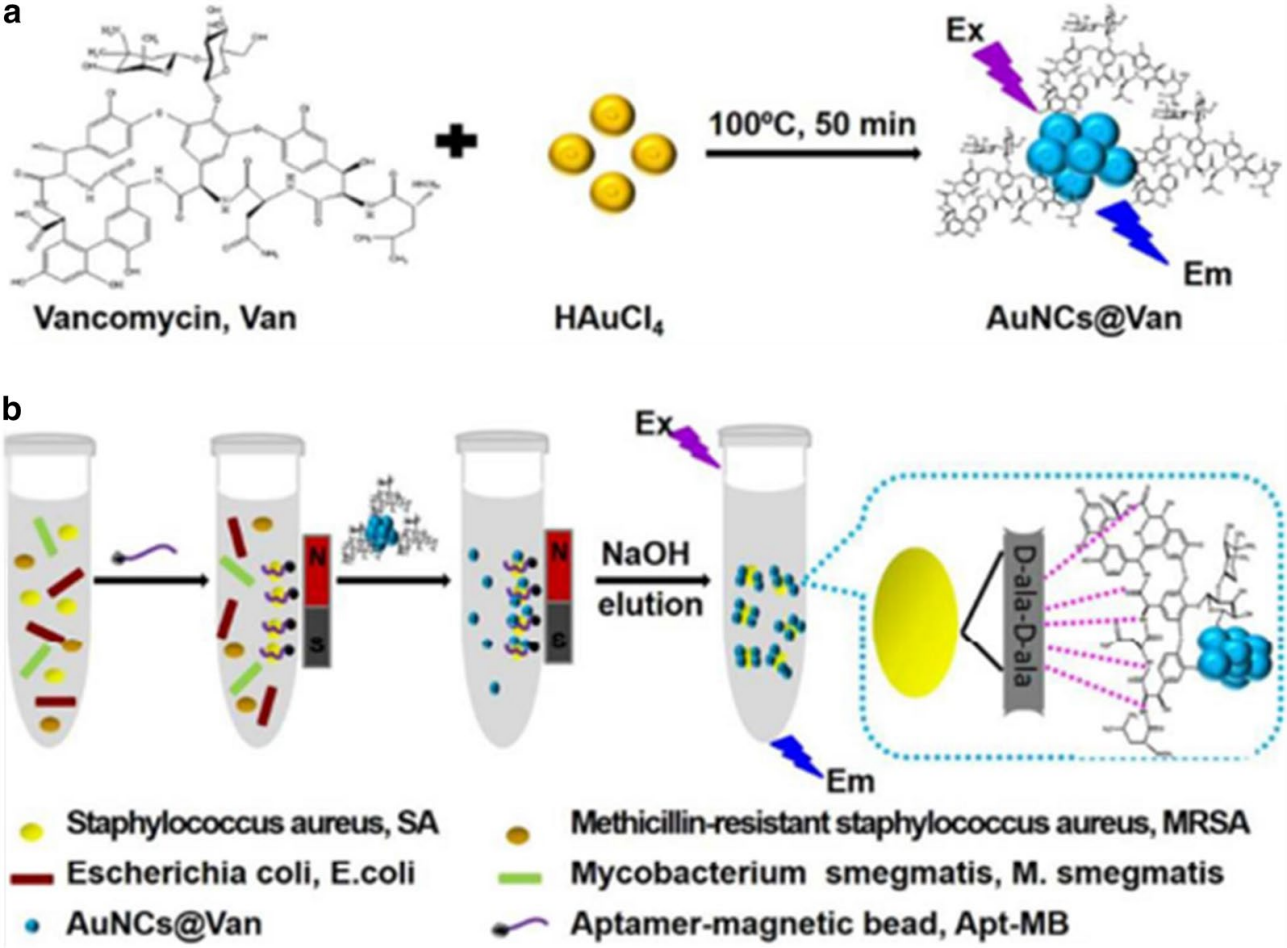
Schematic illustrations of **a** one-step preparation of AuNCs@Van, and **b** determination of SA in mixtures using the Apt-MB and AuNCs@Van dual recognition strategy [113]

Pathogens pose an increasing threat to public health worldwide. It is particularly important to establish a rapid enrichment and sensitive detection method to identify these pathogens. Here we review multiple researchers that have utilized MNPs to detect pathogens. Zhu [109] constructed Fe_3_O_4_/Vancomycin/PEG magnetic nano-carrier, which can efficiently enrich *Listeria monocytogenes* and prepare its nucleic acid in situ for subsequent gene detection (Fig. 1). Chen [110] proposed a magnetic separation (MS) and magnetic relaxation switching (MS-MRS) sensor in which MB250 and MB30 selectively capture and enrich targets to form a “MB250-target-MB30” structure to detect *Salmonella enteritidis* and viruses in milk. This method integrates target enrichment, extraction and detection into one step by using MNPs (Fig. 2). Kwon [111] developed a colorimetric method by combining platinum-coated MNP clusters (Pt/MNCs) with magnetophoretic chromatography to enrich the target with Pt/MNC-EC complex to achieve the naked eye detection of *Escherichia coli* in milk. Wang [112] first synthesized gold magnetic nanoparticles (AuMNPs) core–shell nanocomposite, then synthesized plasma vibrator AunR-DTNB NPs, combined with *staphylococcus aureus* antibody to form a SERS label sandwich structure for enrichment and detection of *Staphylococcus aureus*. Cheng [113] combines aptamer and antibiotic-based dual recognition units with magnetic enrichment and fluorescence detection (Fig. 3), and specifically captures *staphylococcus aureus* with aptamer-coated magnetic beads (Apt-MB). Liu [114] used streptavidin modified MNPs (SA-MNPs) for rapid enrichment and sensitive detection of *Escherichia coli O157:H7*, *Salmonella enteritidis*, *Vibrio cholerae* and *Campylobacter jejuni* in food. Wang [115] proved that Fe_3_O_4_-Ce6-Apt nano-system can identify and enrich blood bacteria, and can detect the enriched bacteria by fluorescence microscopy. Kaur [116] developed a rapid and sensitive method of “Miod”, which includes target bacterial cell enrichment based on MNPs, followed by cell lysis and nucleic acid loop-mediated isothermal amplification (LAMP) with signals measured by an in situ optical detection system to identify positive/negative enteric fever infections.

In addition to detecting gene mutations, the detection of tumor markers plays an important role in the early screening of many cancers. In Wang’s [117] report, using double template MNPs as capture probes with alpha-fetoprotein (AFP) and carcinoembryonic antigen (CEA) as template proteins, which were synthesized by self-polymerization of dopamine (DA) on Fe_3_O_4_ NPs, enriched the targets simultaneously and identified AFP and CEA. Zhao [118] uses magnetic liquid to manipulate particles and cells in a microfluidic system, guiding the movement of particles and cells in a label-free and low-cost manner, and by this method, particles and cells can be enriched. Hong [119] reported multi-functional magnetic nanowires with high density of MNPs and five different types of antibodies (Ab mixture—mPpyNWs), which can enrich cells and monitor captured circulating tumor cells (CTC) in real time by simple colorimetric immunoassay. In previous studies, Xiong’s research group [120] synthesized Fe_3_O_4_ magnetic nanoclusters (MNCs) with high magnetization, uniform size and positive charge. On this basis, biomimetic immune magnetosomes (IMSs) are continually being developed for highly efficient enrichment of CTCs. In Liu’s study [121], magnetic iron oxide NPs were coupled with over-expressed folate receptor (FR) and stably attached to the surface of ovarian cancer (OC) cells, thus making the cells magnetic and enabling nondestructive OC cell enrichment and whole blood detection.

Enrichment of target substances, removal of impurities and purification of detection objects provides great sensitivity and convenience for downstream experiments. Table 2 shows enrichment in target substances using applications mentioned in this paper.

**Table 2:** A summary of enrichment

Magnetic particles	Experimental sample	Detection limit	References
Fe_3_O_4_/Vancomycin/PEG	*L. monocytogenes*	10 cfu/mL	[109]
MBs	*S. enterica*	100 cfu/mL	[110]
Pt/MNCs	*E. coli* O157:H7	10 cfu/mL	[111]
Au@MNPs	*S. aureus*	10 cells/mL	[112]
AuNCs@Van	*S. aureus*	70 cfu/mL	[113]
MNPs	*E. coli* O157:H7, *S. enterica, C. jejuni, V. cholerae*	0.2 cells/mL	[114]
Fe_3_O_4_	*S. aureus*, *E. coli*	10 cfu/mL	[115]
MNPs	*S. typhi*	5 cfu/mL	[116]
Fe_3_O_4_	AFP	0.3 pg/mL	[117]
	CEA	0.35 pg/mL	
MNPs	HCT-116, MCF7	–	[119]
Fe_3_O_4_	MCF-7, HepG2, Caco_2_	–	[120]
IO–FA	SKOV3, A549	(4–10) × 10^6^ cells/L, (3.5–5) × 10^9^ cells/L	[121]

## Nucleic acid detection

The high binding capacity, specificity of binding, and the fast-magnetic response makes MNPs ideal for nucleic acid detection in high-throughput samples requiring fast turn-around time.

### Infectious disease identification

MBs introduce a new technical approach of nucleic acid isolation by coupling with molecules that bind specifically to nucleic acids and can be easily isolated by applying magnetic fields (Table 3).

**Table 3:** A summary of infectious detection

Magnetic particles	Experimental sample	Detection limit	References
MBs	Group B streptococci	1.25 × 10^3^ cfu/mL	[122]
MBs	*S. Enteritidis*	1 cfu/mL	[123]
MBs	CPV-2	3 × 10^4^ copies/mL	[124]
MNPs	H9N2, H1N1, H7N9	2 × 10^−2^ pg/mL	[125]
SiO_2_@ MNPs	HBV, HCV, HIV-1	10, 10, 100 cfu/mL	[126]
MBs	Chagas disease, human brucellosis, bovine brucellosis, foot-and-mouth disease	–	[127]
GMBN	*S. choleraesuis*	5 × 10^5^ cfu/mL	[128]
MBs	*C. difficile*	–	[129]
MBs	*Leishmaniosis*	3.125 × 10^3^ ng/μL	[131]
		1 × 10^3^ cells/mL	
AuMagNBs	Influenza virus A	4.42 × 10^−14^ g/mL	[132]

Parham et al. [122] developed a specific and efficient DNA capture method that is compatible with both PCR and non-amplification detection technologies for Group B streptococci (GBS) identification. Superparamagnetic beads were functionalized with oligonucleotide capture probes of different lengths used to capture GBS genomic DNA (gDNA). Captured DNA was then detected using quantitative PCR. Sequence-specific hybridization capture on magnetic microbeads when combined with target amplification approaches such as PCR or with signal amplification technologies such as polymeric biosensors, could provide a rapid and sensitive method for nucleic acid detection from diverse organic samples. This technology could be combined with sample preparation and detection technologies in a microfluidic system to allow point-of-care testing. Zeinhom [123] developed a novel magnetic nano biosensor, which uses MBs coated with SA and biotin-labeled antibodies against Salmonella enteritidis to capture Salmonella enteritidis in milk, cheese and water, while visual quantitative detection is carried out through signal amplification by horseradish peroxidase (HRP).

Zhuang [124] used a magnetic virus DNA kit to extract DNA/RNA for PCR reaction. Once the PCR products were purified by a MB-PCR purification kit, a fluorescence lateral flow immunoassay was carried out. The study combined both PCR and fluorescence lateral flow immunoassay based on magnetic purification analysis to identify quantitative detection of hepatitis C virus type 2. This scheme can be further applied to clinical analysis. Wu [125] used fluorescent MNPs for simultaneous detection of H9N2, H1N1 and H7N9 avian influenza viruses. The application of the MNPs can capture and separate target chains without sample pretreatment, and single particle counting can be performed by fluorescence. This method has simple signal amplification steps, high detection sensitivity and great potential in early diagnosis of various diseases.

Ali [126] advanced that silica modified MNPs were used to extract virus (HBV, HCV and HIV) nucleic acids and amplify them. MNPs modified by three different probes were incubated with RT-PCR biotin-labeled products in different tubes to capture their HBV, HCV and HIV sequences respectively. Finally, 3-(2′-spiroadamantane)-4-methoxy-4-(3′-phosphoryloxy) phenyl-1,2-dioxetane was added and chemiluminescence (CL) was recorded. This method can achieve high throughput, automation and simultaneous detection of multiple nucleotide sequences.

María designed a biosensor based on electrochemical magnetic microbeads [127]. The platform is portable, durable and inexpensive. Experiments have verified its detection function in on-site diagnosis of human (Chagas disease and human brucellosis) and animal (bovine brucellosis and foot-and-mouth disease) infections. This platform technology can also be used to diagnose other infectious and non-infectious diseases. In Xia’s research [128], gold magnetic bifunctional nanobeads (GMBN) were used to prepare immunochromatographic test strips for the detection of Salmonella choleraesuis. The immunochromatographic test strip can be applied to food samples such as whole milk. In their research, Jacquelyn [129] proposed to use the combination of MB aggregation and microfluidic manufacturing technology to detect pathogens. In this experiment, the LAMP product was coupled with the product inhibition bead aggregation and used to detect the target sequence of Clostridium difficile. Chen [130] designed a new point-of-care test (POCT) system based on MNPs. After testing the performance of the instrument, an automatic real-time adenovirus detection experiment was carried out. The experimental results are similar to those of commercial systems, proving the reliability of the system. Margarita [131] used MB capture probes to separate Leishmania specific surface antigen and DNA from the solution for cadmium selenite quantum dot detection. These methods have great potential in clinical application of human and veterinary medicine.

Recently, Sangjin [132] combined magnetic nano-beads (MagNBs) and gold nano-particles in silica shell to carry out enzyme-linked immunosorbent assay to monitor, reduce the spread, and provide immediate clinical treatment to influenza A virus. Feng’s [133] liver samples treated with MBs were tested for *Yersinia pestis* by LAMP. The combination of MBs and LAMP is a simple, rapid and sensitive detection method, suitable for field or poor areas. Fang [134] designed a high flux magnetic separation module and a heating and vibration module, which can collect a large number of purified nucleic acids from pathogens of infectious diseases. In the experiment, the system is applied to nucleic acid extraction of human whole blood, and has the capability of high-throughput sample preparation to diagnose infectious diseases.

### Mutation site detection

Single nucleotide polymorphism (SNP) is the most common variation in the human genome. The study of SNPs helps to explain the phenotypic differences of individuals, the susceptibility of different groups and individuals to complex diseases, their tolerance to various drugs, and their responses to environmental factors [135]. Some SNPs are mutation sites of disease-causing genes, which have practical research significance and application prospect in various aspects such as susceptibility assessment, early diagnosis, prevention and treatment of diseases [136, 137]. Due to the special physicochemical properties and biocompatibility of MNPs such as, high separation speed, high efficiency, reusability, simple operation, no need of expensive instruments and no influence on the activity of separated substances, they have been widely used in various biological detections.

Early on, Mirkin’s group [138] used short DNA modified NP labeled with hundreds of biological barcoded DNA (Bbc-Au NP) as a signal probe and another DNA modified magnetic microparticle (Oligo-MMP) as a capture probe to form a Bbc-Au NP/DNAtarget/Oligo-MMP hybrid structure after complementary pairing. The unlabeled detection and SNP detection of the target DNA can be detected at the same time. Ngo [139] used a sandwich hybridization method to detect a specific DNA sequence of plasmodium falciparum, and distinguished SNPs of wild-type malaria DNA and mutant malaria DNA. The method used loading report probe on ultra-bright SERS nanorattle, while using a magnet to concentrate the hybridization sandwich for SERS measurements. This scheme is simple and convenient and has great potential for integration into portable instruments, although is limited by the number of samples.

Based on the shortcomings mentioned above, Liu [140] designed a high-throughput SNP genotyping based on solid-phase PCR on MNPs with dual-color hybridization for detection the C677T polymorphisms of methylenetetrahydrofolate reductase (MTHFR) gene. This method solves the waste of nanoparticles. Using the PMMA/Fe_3_O_4_ nanocomposite particles synthesized by Wang [141] for the SNP genotyping, the PCR reaction was directly carried out on the surface of streptavidin-coated magnetic nanoparticles SA-MNPs. The results showed that the dual-color probes have three fluorescence patterns on the microarray, differentiating three different genotypes. All reactions can be carried out in the same container without any purification, concentration and other processes [142]. This is a simple and labor-saving SNP genotyping method, and the typing result is intuitive and accurate. It can be applied to automated systems to realize high-throughput SNP detection. However, due to the need to design a pair of special two-color fluorescent probes for each SNP site to be analyzed, this method is relatively expensive. Tang [143] uses the same method as the above principle, with MNPs to carry out PCR and dual-color fluorescence hybridization to detect SNP sites in Pseudomonas aeruginosa exoS gene.

With deepening research, Li [144] designed a high-throughput, automated SNPs genotyping method based on AuMNP arrays and dual-color single base extension. The advantage of this assay is that it does not need to carry out necessary procedures for purification and complex reduction of PCR products, simplifying the process and increasing the potential for automation. Firstly, the biotin-labeled primer was captured by SA-GMNPs, the ssPCR product was hybridized with the primer to carry out dual-color single base extension, the fluorophore-GMNP complex of each sample was fixed on the slide, and the genotype of each sample was determined by measuring the fluorescence intensity of the fluorophore-GMNP complex on the array. Li [145] then designed a universal label for high throughput SNP genotyping to detect C667T, A1298C, M235T, G93A. The universal tag probe sequence is divided into three parts, one end of which is an allele-specific probe, and the middle of which is connected with 11 bases of polyT. The other end is connected with Cy3 or Cy5 labeled universal detector. The genotype of the sample is distinguished by the detection of fluorescent signals. The universal label probe technology is applied to detect the typing of multiple SNP sites by using a pair of fluorescent labeled probes, thus greatly reducing the typing cost.

**Fig. 4:**
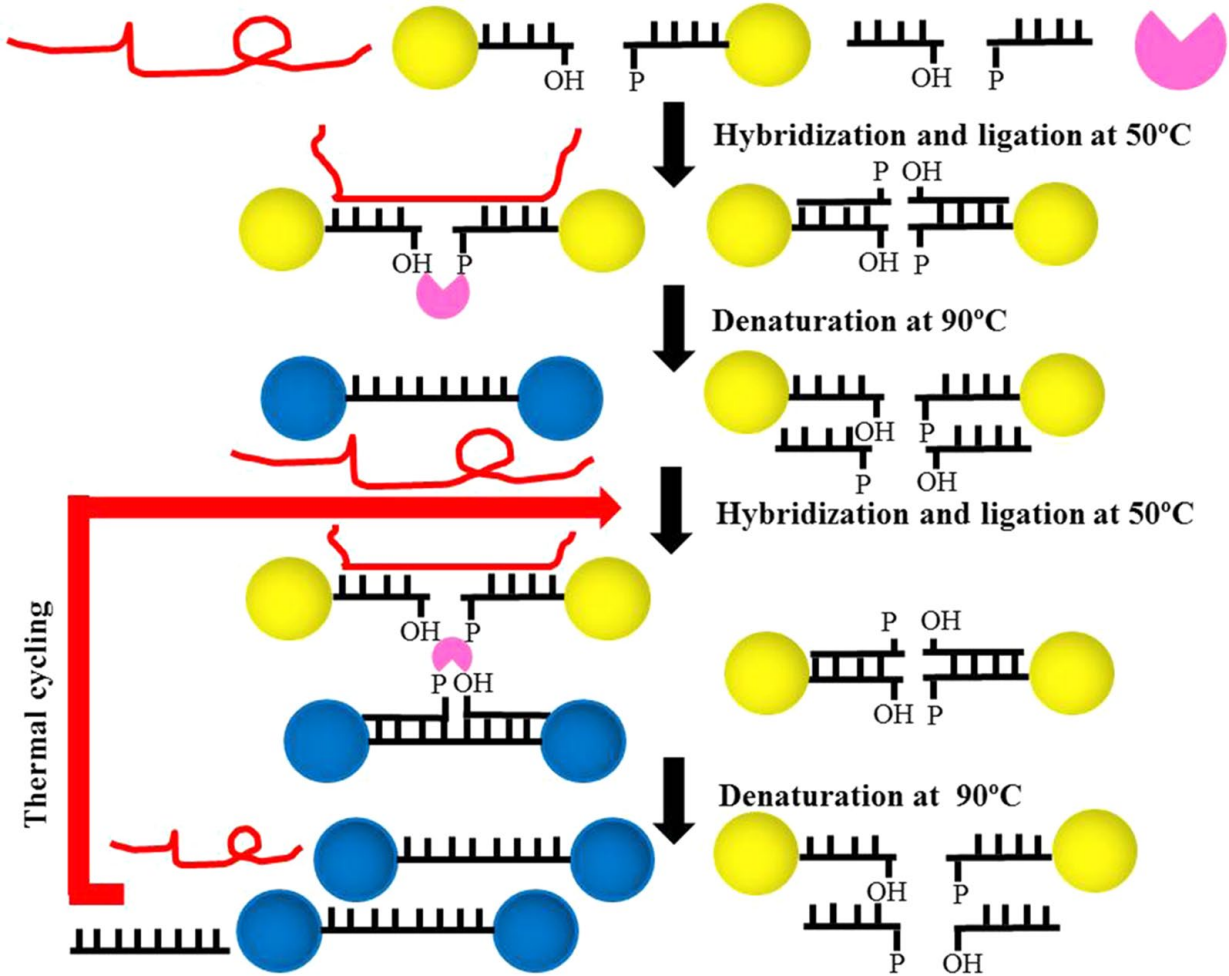
Schematic illustration of the real-time LCR DNA assay [146]

AuNPs make ligase chain reaction a simple SNP genotyping method. Shen [146] prepared two kinds of AuNPs coated with capture probes. The correctly paired target DNA hybridized with it’s complementary probe, then heated up to denature all the double-stranded structures formed, followed by exponential amplification, resulting in the formation of AuNPs aggregates whose color changed from red to purple/gray (Fig. 4). Min [147] proposed to use *ExoIII* nuclease and MNPs for SNP typing and detection, which use double-probe detection system with signal amplification. This method amplifies the detected fluorescence signal by 6063 times and has high selectivity and sensitivity. Yan [148] used the PCR-gold magnetic lateral flow assay system (PCR-GoldMag LFA) to make the SNP typing results visible to the naked eye without the need for other signal collectors.

In another report, Hui [149] reported for the first time that the system of combining amplification refractory mutation system (ARMS)-PCR with GoldMag based lateral flow analysis (LFA) for genotyping was applied to the genotyping of MTHFR C677T. PCR-LFA can be extended to detect other SNPs of genes related to disease risk, drug metabolism or drug response.

Previously, in Liu’s [150] research, we reported a sensitive, low-cost and easy-to-use POCT system, which was formed by combining ARMS PCR with GMNPs and LFA called ARMS-LFA system. This method simultaneously used uniform conditions to detect multiple SNPs. In this study, 7 pathogenic SNPs in phenylalanine hydroxylase gene were genotyped.

DNA methylation is the earliest found relation to gene silencing in epigenetic mechanisms [151–153]. This refers to the process of adding methyl groups to the 5c end of cytosine in cytosine-guanine dinucleotides (CpG sites) to convert cytosine into 5-methylcytosine [154, 155]. Research shows that DNA methylation modification can regulate the expression of specific genes through a variety of ways, including affecting the development process of cells and maintaining cell stability. Once methylation modification appears abnormal, it can cause the occurrence of differing diseases [156, 157].

**Fig. 5:**
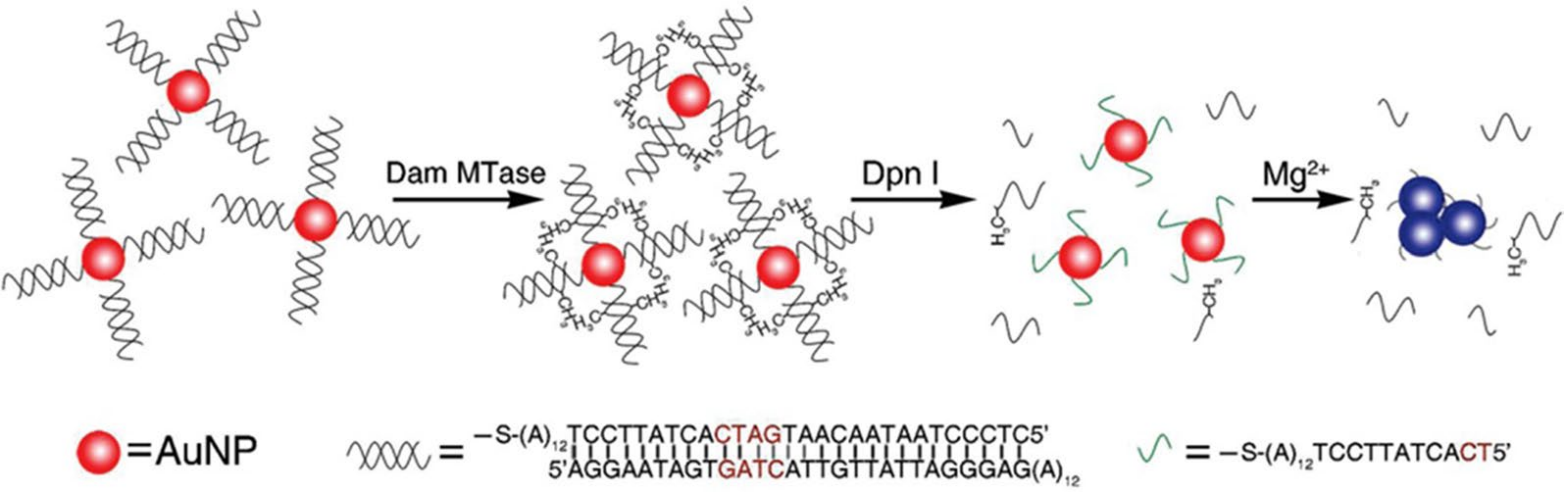
Schematic illustration of the proposed strategy for methylation detection [158]

Liu [158] used DNA modified AuNPS combined with an enzyme linked reaction to monitor DNA methylation. The double-stranded DNA molecules modified on the surface of AuNP were first methylated by DNA adenine methylation (Dam) methyltransferase and then cleaved by methylation sensitive restriction endonuclease DPN I. Removing double chains from the surface of AuNP through methylation/cleavage process will make the NPs unstable, resulting in aggregation of AuNP and color change from red to blue. Therefore, the enzyme activity of dam MTase and DNA methylation can be detected (Fig. 5).

Ge [159] used a method based on the color change of solution caused by AuNP enrichment to observe and detect DNA methylation. The probe was released by thermal denaturation and added to the unmodified AuNP solution. Salt induced aggregation was used for colorimetric detection. Compared with other methods using sodium bisulfite and methylation-specific restriction enzymes, this method provided a simpler rapid DNA methylation detection.

Through a new HCR-BRCA strategy, Bi [160] developed a CL method for methylation detection and MTase activity determination to improve sensitivity. MNPs separation, reaction and detection were used. The experiments were carried out under their respective optimal conditions. This method has advantages of specific recognition of MTase and methyl sensitive endonuclease, high RCA amplification efficiency and good MNPs control, although, the current analysis steps are complicated.

Dadmehr [161] developed a very sensitive and convenient fluorescent biosensor based on Fe_3_O_4_/Au core/shell NPs for rapid detection of DNA methylation. After the target unmethylated and methylated ssDNA was added, the fluorescence intensity increased linearly with the increase of unmethylated ssDNA concentration. This detection system will be applied to the early diagnosis of methylation-dependent diseases such as cancer.

### NGS library preparation

MNPs are widely used in high-throughput sequencing for library preparation, purification, concentration of PCR products and subsequent nucleic acid sequencing. The magnetic target purification method allows for target purification directly from the PCR reaction. This purification removes excess salts, enzymes, primers and nucleotides through a simple washing procedure, which eliminates the need for centrifugation steps thus simplifying the purification procedure.

Zhang [162] used MNPs to separate plasma DNA and prepare plasma DNA libraries. Short cell-free mitochondrial (cf-mt) DNA fragment was obtained by using size selection to separate out the shorter DNA fragments. This method was applied to plasma samples of septic patients and the heterogeneity of mtDNA in plasma of 3 patients was detected. This study is of great significance to the importance of MNPs in the discovery of the architecture of cfDNA and their biomarkers. Fadeev [163] explored sequencing the genome of *Alteromonas* HOT1A3. MBs enriched PCR products in this experiment and allowed this group to discover a previously undiscovered 148kbp plasmid.

Inflammatory bowel disease (IBD) is a chronic gastrointestinal inflammatory disease. Hall’s [164] team performed a genome sequencing of fecal samples from 20 IBD patients and 12 controls. Total nucleic acids were extracted with MBs. Sequencing allowed this group to study the IBD gut microbiome at the highest resolution of their functional potential. In Schoenfelder’s research [165], mammalian gene expression control was learned through high-throughput sequencing. High-throughput sequencing has also helped us understand the correlation between human genetic diseases and the identification of underlying disease genes.

To increase throughput for NGS, MNPs are widely used to process samples. Wu et al. [106] completed the preparation of 96-well plate samples with MNPs, meeting the needs of high-throughput sample preparation. Baker [166] prepared NGS libraries for constructing CFTR gene amplicon library using MiSeqDx IUO CF analysis reagent (Illumina), and used MBs for normalizing the libraries prior to pooling. The libraries were sequenced and were screened for neonatal CF. Hertz [167] uses MNPs and the Illumina MiSeq platform to examine pathogenic mutation genes related to sudden infant death syndrome.

Franasiak [168] sequenced 16S ribosome subunits of endometrial microflora during embryo transfer; Kostareva [169] analyzed 24 cases of idiopathic restrictive cardiomyopathy including 108 cardiomyopathy and arrhythmia related genes; Kou [170] carried out sequencing experiments on genes related to simulated samples in order to detect low abundance bladder cancer cells carrying a large number of mutations from urine;. Fisher [171] conducted clinical tests on somatic cell variations of non-small cell lung cancer, melanoma and gastrointestinal malignant tumors; Weimer [172] put forward human leukocyte antigen typing and sequencing which improved the overall survival rate of patients after hematopoietic stem cell transplantation; He [173] used the technology to improve the screening of thalassemia carriers among premarital adults in high-prevalence population; Operario [174] used amplicons to test the drug sensitivity of Mycobacterium tuberculosis; Dirani [175] uses next generation sequencing technology to genotype hepatitis C virus for clinical treatment, which is one of the major causes of liver-related death; Arias [176] tests HIV virus in humans through NGS and monitors drug resistance mutation, providing information on low-frequency drug resistance mutation and virus distribution in blood, so as to make sustainable response to treatment and better disease management. All of these researchers used MBs or MNPs for library preparation for NGS.

In nucleic acid detection, the traditional method of extracting and detecting nucleic acid has lower efficiency, complex protocols, expensive instrumentation and requires highly skilled staff. Therefore, the automation of experiments has attracted great attention of researchers. Using magnetic particles to develop automatic nucleic acid detection technology [177], greatly simplifies the experimental process and improves the experimental efficiency and accuracy.

Chen [178] developed a nucleic acid separation and purification instrument using MNPs, including three modules of temperature control, up-and-down movement and computer software, which are used in the whole process of automated extraction of nucleic acid from *Escherichia coli* O157: H7 for rapid screening of foodborne pathogens. Similarly, Ali designed a virus nucleic acid extraction scheme. The semi-automatic system is used to detect hepatitis B virus and hepatitis C virus simultaneously. Chen [107] designed the scheme of nucleic acid purification technology and its application on the basis of automatic extraction of nucleic acid, and then isolated and purified the sample of hepatocellular carcinoma, and obtained high-yield and high-purity nucleic acid through experiments. After automation is realized, Wang’s [179] proposal can process multiple samples at the same time for multi-channel detection, with simple operation and greatly improved detection efficiency.

With the deepening of research, Chen [130] proposed a POCT system using MNPs that automated real-time testing for pathogens. Automated nucleic acid extraction had a higher repeatability and performed better when compared to manual testing. In recent years, many researchers such as Verigene (Nanosphere, Northbrook, US), iCubate2.0 (iCubate, Huntsville, US) and Rheonix (Rheonix, Ithaca, US) have used gene chips as detection tools and integrated microarrays into automated diagnostic equipment [180–183]. With the help of MNPs, these devices can automatically amplify, hybridize and detect gene targets.

### Other applications

Liu [184] used MNPs to develop a new method using ligation-dependent PCR, and CL detection technique for quantitative analysis of copy number polymorphisms; however, this method needs improvement for both stability and repeatability. Hoan [185] used Raman Scattering for their study. A sandwich hybridization with MBs was used for nucleic acid detection [186, 187] with target sequences and ultra-bright SERS nanorods, while also identifying SNPs. The detection scheme includes sandwich hybridization of MBs bound to the capture probe, target sequences, and ultra-bright SERS nanorods bound to the report probe. After hybridization, the sandwich probe is concentrated at the detection focus controlled by the magnetic system for SERS measurement. Li [188] proposed a new signal amplification strategy for double detection of MEG3 specific sequences using MNPs. This method was able to detect DPV values of Fc and MB as low as 1fM to 100 pM. Genetic sensors have high selectivity and repeatability and have the potential to be a powerful tool for multiple quantitative analysis in early clinical diagnosis.

In Amini’s research [189], two kinds of NPs were designed and utilized. The first kind was AuNPs wrapped by probe 1 and bio-barcoded DNA. The second kind was MNPs wrapped by probe 2. The two kinds of functionalized NPs were mixed, and then gDNA was added into the solution to react, forming MNP-probe2-genomic DNA-probe1-AuNPs-bio-barcode DNA complex, washing out unreacted impurities. Then DTT was used to release the bio-barcode DNA, and fluorescence spectrophotometry was used to analyze the supernatant containing the bio-barcode DNA. Researchers used this method to detect *Staphylococcus aureus*.

He [190] proposed a method for detecting circulating miR-142-3p in human serum by using functionalized MNPs. MNPs were used as the capture nanoprobes, and poly-HRP were used as the signal amplification probes. HRP labeled DNA H1 and H2-1 self-assembled to form poly-HRP. Streptophilin-modified MNPs were added to the lysate, with biotin-labeled capture genes attached to its surface, poly-HRP probe and auxiliary DNA were added. After hybridization, the CL signals were significantly amplified. MiR-142-3p of human serum sample is taken as an experimental sample to verify the feasibility of the method.

In order to diagnose neurodegenerative diseases of fragile X syndrome, Ren [191] loaded SA on MNPs, and then attached DNA modified with biotin as a capture probe. Target DNA hybridized with probe DNA and short report DNA labeled with carboxy-fluorescein (FSR-DNA) to form complexes respectively. After the reaction was completed, FSR-DNA supernatant released was subjected to fluorescence detection through magnetic separation and de-hybridization. The method is simple to operate and provides great expectations for early diagnosis of the disease.

Tian [192] used DNA-based HCR and MNPs coupled with Fe_3_O_4_ to detect miRNA. Copper (II) complex was used as a small molecule enzyme mimic for miRNA signal while Fe_3_O_4_ were used as a separate target under magnetic field and was able to detect miRNA sensors. This method is expected to become a scheme to study next generation microRNA sensors, without the need of enzyme labeling or fluorophore labeling.

P53 is a human tumor suppressor gene, which is of great significance to human health. Therefore, Wang [193] established a CL method to detect the gene and used a new type of efficient luminescent material, 2′,6′-dimethylcarbonylphenyl-10-sulfopropyl acridinium-9-carboxylate 4′-NHS ester (NSP-DMAE-NHS). The method comprises the following steps: firstly coating BSA on AuMNPs, and then connecting amino wild-type p53 genes. The functionalized MNPs were mixed with ssDNA-(NSP-DMAE-NHS) to hybridize. After the reaction is completed, unreacted impurities are removed by magnetic separation, then HNO_3_ + H_2_O_2_ and NaOH are added to trigger CL, and the luminous intensity is detected by a measuring system. The method can also be used for early diagnosis and treatment of cancer.

Wen [194] designed a convenient and quick method to capture and release hepatitis B virus DNA. Biotinylated captured DNA sequence of HBV was attached to MNPs coupled with SA, while the unwanted portion was blocked with biotin and bovine serum protein. Biotinylated target DNA sequence of HBV, hybridized and paired with the previously functionalized MNPs, the reaction compound is magnetically separated and then incubated with SA conjugated CdSe/ZnS quantum dots, unreacted impurities are washed away, and supernatant is obtained after de-hybridization at high temperature for fluorescence determination. The method has been directly applied to the detection of serum samples, with good experimental results and practical application potential.

## Conclusion

In this review, we have shown the application of MNPs in nucleic acid extraction, target substance enrichment and nucleic acid detection. We summarized the sequencing methods of DNA, SNP and methylation genes. In January 2003, the completion of human genome project led to several important findings related to the structure of the human genome [195, 196]. SNP and methylation are included in this study’s findings, which we reviewed in this paper. SNP and methylation are two forms of gene mutation, which are closely related to diseases [197–202]. In-depth research was conducted on SNP and methylation to find new courses for early detection of cancer [203–206]. The simultaneous detection of SNP and methylation at multiple sites by MNPs is conducive to the early diagnosis and prevention of cancer [207–210]. MNPs are widely used [211–215] because they are biocompatible and easily separated. The application of NPs is increasingly important in biotechnology. The MNPs used in this paper, like Fe_2_O_3_/Fe_3_O_4_, Fe_3_O_4_/Au, SiO_2_/Fe_3_O_4_, and Fe_3_O_4_ combined with PCR-assisted methods, radio-labeling techniques [216], HPLC [217], gel electrophoresis [218] and alternative approaches such as fluorescent, colorimetric, electrochemical and CL simplifies the experimental steps and improves the sensitivity and specificity of detecting DNA sequences, SNPs and methylation.

The birth of high-throughput sequencing technology is a milestone event in the field of genomics research [219–225]. This technology by chip which read sequencing at millions of points at the same time makes the single base cost of nucleic acid sequencing increasingly lower than that of the first-generation sequencing technology. Taking human genome sequencing as an example, the human genome project carried out at the end of last century cost 3 billion US dollars to decode the human life code, while the second-generation sequencing has brought human genome sequencing into the era of meta-genomes. Such a low cost of single-base sequencing enables us to implement genome projects of more species and thus decrypt the genetic codes of genomes from more biological species. At the same time, among species that have completed genome sequencing, it is also possible to carry out large-scale whole genome resequencing [226, 227].

Genes are the biological genetic information base, which supports the basic structure and performance of life and stores all the information of life’s race, such as blood type, gestation, growth, apoptosis and other processes [228–232]. The change of a base in a DNA sequence affects human health. Therefore, it is of great significance to develop rapid, real-time and accurate sequencing methods. Due to the complexity of human blood samples and the possibility of suffering from different diseases at the same time, there is an urgent need to develop a method to detect many different diseases in a single diagnosis [233–239]. The application of nano-magnetic material-based high-throughput sequencing continuously improves the sensitivity, selectivity and detection sample size, which meets the above requirements [240–242]. The selection of magnetic nano-materials with large specific surface area and easy separation can be combined with various detection methods to obtain better sensitivity, selectivity and simultaneous detection of different sequences [243–253]. However, we still need further in-depth and detailed research in order to obtain a simpler and more flux sequencing method.

Although MNPs have many advantages, improvements are still needed in many aspects. Firstly, the magnetic separation performance of MNPs should be improved, thus meeting the application requirements in fast response and accurate positioning under external magnetic field [254, 255]. Secondly, MNPs stability could be improved, because it directly affects its application, for example, storage stability could be improved so that MNPs can be shipped and stored at room temperature [256]. Finally, preparation of MNPs with multi-function improves surface functionalization and ligand bonding efficiency [257].

MNPs combined with molecular diagnostic systems are used to develop fully-automatic and closed nucleic acid detection assays. If detection time and sensitivity can be improved, it will have great impact in clinical detection, biomedicine and other fields.

## Data Availability

Not applicable.

## Abbreviations

NPs: Nanoparticles
MNPs: Magnetic nanoparticles
MRI: Magnetic resonance imaging
LOC: Lab-on-a-chip
AML: Acute myeloid leukemia
PCR: Polymerase chain reaction
MBs: Magnetic beads
SERS: Surface-enhanced Raman scattering
PAD: Paper-based analytical device
HCR: Hybridization chain reaction
SNP: Single nucleotide polymorphism
MS: Magnetic separation
MS-MRS: Magnetic relaxation switching
Pt/MNCs: Platinum-coated MNP clusters
AuMNPs: Gold magnetic nanoparticles
SA-MNPs: Streptavidin modified MNPs
LAMP: Loop-mediated isothermal amplification
AFP: Alpha-fetoprotein
CEA: Carcinoembryonic antigen
DA: Dopamine
CTC: Circulating tumor cells
MNCs: Magnetic nanoclusters
IMSs: Immune magnetosomes
FR: Folate receptor
OC: Ovarian cancer
GBS: Group B streptococci
gDNA: Genomic DNA
HRP: Horseradish peroxidase
CL: Chemiluminescence
GMBN: Gold magnetic bifunctional nanobeads
POCT: Point-of-care test
MagNBs: Magnetic nano-beads
Bbc-Au NP: NP labeled with hundreds of biological barcoded DNA
Oligo-MMP: DNA modified magnetic microparticle
MTHFR: Methylenetetrahydrofolate reductase
MEM-PCR: Magnetic enrichment-multiplex PCR
ARMS: Amplification refractory mutation system
LFA: Lateral flow analysis
Dam: Adenine methylation
Dam: Adenine methylation
APC: Adenomatous polyposis coli
MMPs: Magnetic microspheres
ssDNA: Single-stranded DNA
cf-mt: Cell-free mitochondrial
IBD: Inflammatory bowel disease
NGS: High-throughput sequencing
FSR-DNA: Short report DNA labeled with carboxy-fluorescein
NSP-DMAE-NHS: 2′,6′-dimethylcarbonylphenyl-10-sulfopropyl acridinium-9-carboxylate 4′-NHS ester
